# Factors predicting final visual outcome in quiescent proliferative diabetic retinopathy

**DOI:** 10.1038/s41598-020-74184-9

**Published:** 2020-10-14

**Authors:** Jinsoo Kim, In Won Park, Soonil Kwon

**Affiliations:** Department of Ophthalmology, Hallym University Sacred Heart Hospital, Hallym University College of Medicine, 22, Gwanpyeong-ro 170beon-gil, Dongan-gu, Anyang, 14068 Republic of Korea

**Keywords:** Medical research, Risk factors

## Abstract

To investigate factors reflecting visual outcome and macular perfusion in quiescent proliferative diabetic retinopathy (PDR) patients after panretinal photocoagulation (PRP). We included 118 patients with quiescent PDR who had completed PRP. All participants had standardized interview to determine ocular history, smoking status, cardiovascular risk factors, and history of diabetic mellitus (DM). Foveal avascular zone (FAZ) area, retinal vessel density (VD) and vessel length density (VLD) were measured using optical coherence tomography angiography. VD was negatively correlated with hypertension, diabetic foot, HbA1c, and time after PRP (β =  − 0.181, *P* = 0.046; β =  − 0.231, *P* = 0.020; β =  − 0.244, *P* = 0.010; β =  − 0.278, *P* = 0.029). FAZ area of superficial capillary plexus and deep capillary plexus (DCP) was positively correlated with DM duration and diabetic foot (β = 0.178, *P* = 0.047; β = 0.293, *P* = 0.002; β = 0.252, *P* = 0.045; β = 0.304, *P* = 0.002). Macular perfusion state in patients with quiescent PDR was associated with diabetic foot, DM duration, HbA1c, and time after PRP. Of note, diabetic foot showed the strongest correlation with macular perfusion among various systemic factors. VLD, especially in DCP was associated with poor visual outcome.

## Introduction

Diabetic retinopathy (DR) is the most common microvascular complication of diabetes mellitus (DM) and the leading cause of blindness in working-aged adults worldwide^1–4^. Chronic hyperglycemia in DM causes damage to capillaries, resulting in retinal ischemia and increased vascular permeability. The resulting DR progresses from nonproliferative diabetic retinopathy (NPDR) to proliferative diabetic retinopathy (PDR), which is a stage that can lead to the formation of retinal neovascularizations. PDR can progress to vitreous hemorrhage or tractional retinal detachment and, finally, blindness^4,5^.

Panretinal photocoagulation (PRP) has been the main treatment to prevent severe vision loss in patients with PDR since its introduction by The Diabetic Retinopathy Study in the late 1970s^6^. However, patients with PDR who complete PRP show a variety of visual outcomes, including severe loss of vision.

Some studies of long-term visual outcomes in DR after PRP have been reported. The Early Treatment Diabetic Retinopathy Study (ETDRS) reported that 42% of patients had visual acuity of 20/20 or better in the better eye, and 84% had visual acuity of 20/40 or better in the better eye. Compared with baseline, 20% of patients had moderate vision loss in the better eye at follow-up, and only 1% had severe loss at 5 years^7^. The known factors associated with long-term visual outcomes in DR include regular ophthalmic follow-up, control of blood sugar and lipids, blood pressure, visual acuity at baseline, and presence of neovascularization of the disc^7–10^. Not surprisingly, macular perfusion status is one of the most important factors that determines the visual prognosis in patients with DR^11^. However, limited information on the long-term change of macular perfusion after PRP is available in the literature.

Macular perfusion status can be evaluated easily with recently developed optical coherence tomography angiography (OCTA). OCTA is a noninvasive imaging technique that can visualize the retinal and choroidal vasculature down to the capillary level in finely divided tissue slabs^12^. Quantitative data, such as the foveal avascular zone (FAZ) area, capillary nonperfusion area, and vessel density (VD) can be easily obtained by using OCTA^13^. Many studies that used OCTA have shown an association of visual acuity with OCTA parameters^11,13–17^. Some studies of DR have reported a deterioration of OCTA parameters as the stage of DR progresses^17–21^. In addition, studies of macular perfusion status after PRP have been actively reported^22–25^; however, the sample sizes were relatively small and the follow-up periods were short in most studies. To the best of our knowledge, no previous study has investigated the relationship between OCTA parameters and systemic factors in patients with DR. Accordingly, we aimed to use OCTA in a study of visual outcomes and macular perfusion status in patients with quiescent PDR after PRP and investigate their association with systemic factors.

## Results

### Patient characteristics

A total of 60 men and 58 women with a mean age of 58.9 ± 10.9 years were included in this study. Hypertension (61.9%) was the most common comorbid disease, followed by dyslipidemia (45.8%). The mean duration of DM was 17.1 ± 10.3 years. Thirteen patients (11.0%) were receiving hemodialysis for diabetic nephropathy, and 15 patients (12.7%) were suffering from diabetic foot. The average HbA1c was 7.27 ± 1.10% (Table 1).

**Table 1:** Demographics, diabetes status, and systemic characteristics of patients.

	N = 118 patients
Age (year, mean ± SD, range)	58.9 ± 10.9 (31–85)
Gender, male, n (%)	60 (50.8)
**Medical history**	
Smoking status (current/never/past)	16/72/30
Hypertension, n (%)	73 (61.9)
Dyslipidemia, n (%)	54 (45.8)
History of CVA, n (%)	12 (10.2)
History of IHD, n (%)	17 (14.4)
BMI (kg/m^2^)	24.5 ± 4.5
**Diabetes history**	
Diabetes duration (year, mean ± SD, range)	17.1 ± 10.3 (1–46)
Medication use (no/oral/insulin/oral with insulin)	2/67/32/17
Hemodialysis, n (%)	13 (11.0)
Diabetic foot, n (%)	15 (12.7)
HbA1c, % (mean ± SD)	7.27 ± 1.10

*SD* standard deviation, *CVA* cerebrovascular accident, *IHD* ischemic heart disease, *BMI* body mass index.

The average best corrected visual acuity (BCVA) was 0.179 ± 0.199 logMAR (logarithm of the minimum angle of resolution). The average time after completion of PRP was 7.1 ± 5.4 years, and the average number of intravitreal anti-vascular endothelial growth factor injections was 0.6 ± 1.4. Among 118 eyes, 58 eyes (49.2%) were pseudophakic, and 33 eyes (28.0%) had undergone vitrectomy. The mean central foveal thickness (CFT) was 240.9 ± 46.2 μm (Table 2).

**Table 2:** Ocular history and central foveal thickness of patients.

	N = 118 eyes
BCVA (logMAR, mean ± SD)	0.179 ± 0.199
Time after PRP (y, mean ± SD, range)	7.1 ± 5.4 (1.0–20)
Anti-VEGF injections (mean ± SD, range)	0.6 ± 1.4 (0–9)
Pseudophakia (%)	58 (49.2)
Vitrectomy, n (%)	33 (28.0)
CFT (μm, mean ± SD)	240.9 ± 46.2

*BCVA* best corrected visual acuity, *logMAR* logarithm of the minimum angle of resolution, *SD* standard deviation, *PRP* panretinal photocoagulation, *VEGF* vascular endothelial growth factor, *CFT* central foveal thickness.

### OCTA parameters of retinal layers and zones

**Figure 1:**
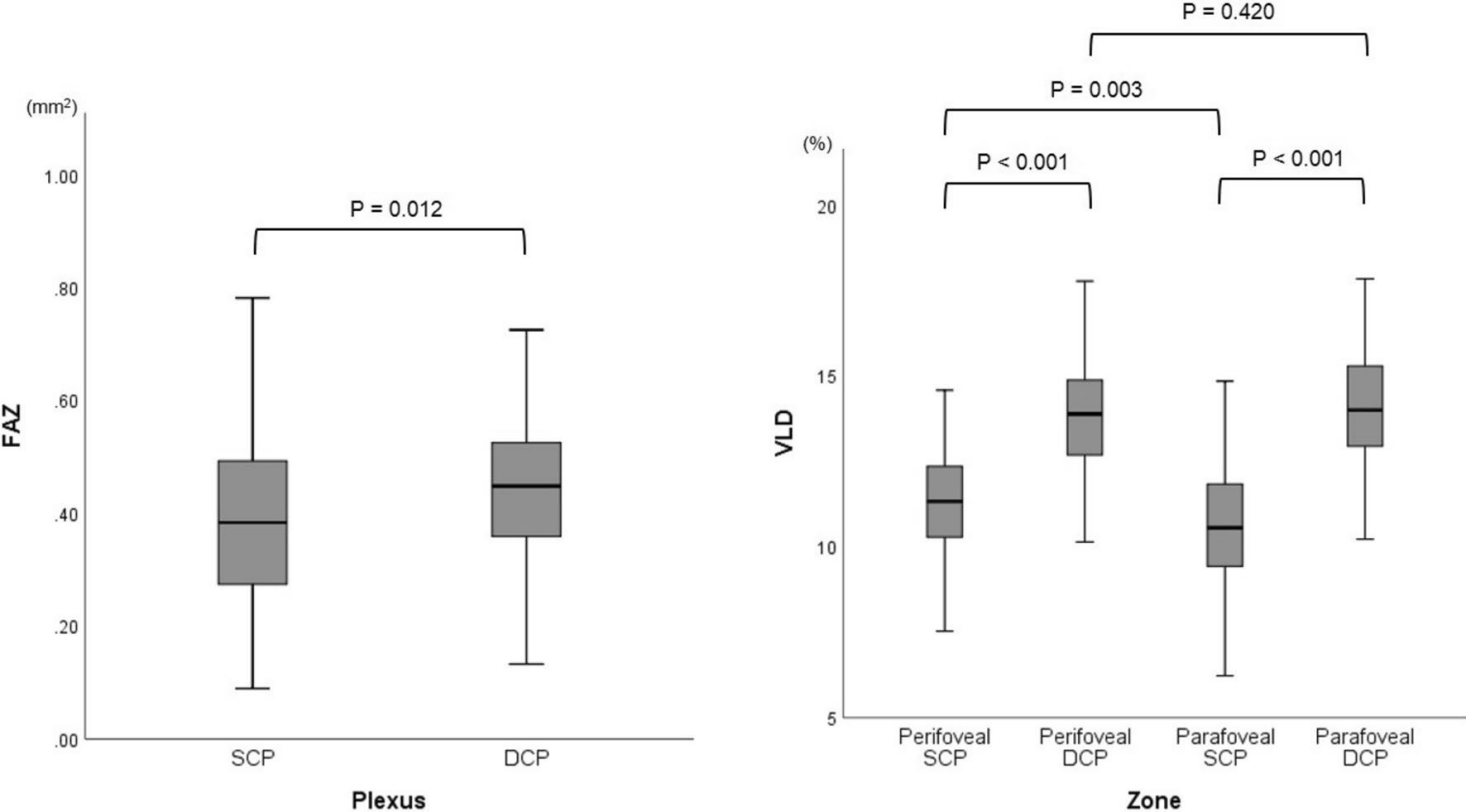
Foveal avascular zone area (**A**), and vessel length density (**B**) in patients. The boxes contain values between the 25th and 75th quartiles; the central lines represent the median values; Whiskers above and below the box indicate the 90th and 10th percentile. Independent *t* test was used to compare FAZ, and VLD. *SCP* superficial capillary plexus, *DCP* deep capillary plexus, *FAZ* foveal avascular zone, *VLD* vessel length density.

The mean FAZ area of the superficial capillary plexus (SCP) was 0.44 ± 0.28 mm^2^, and the mean FAZ area of the deep capillary plexus (DCP) was 0.51 ± 0.36 mm^2^. The difference of the FAZ area between the SCP and DCP was statistically significant (*P* = 0.012). The mean vessel length density (VLD) was 11.3 ± 1.6% in perifoveal SCP, 13.8 ± 1.7% in perifoveal DCP, 10.6 ± 1.9% in parafoveal SCP, and 14.0 ± 1.9% in parafoveal DCP. The VLD of perifovea and parafovea showed significant difference in the SCP and DCP (*P* < 0.001, and *P* < 0.001, respectively). In addition, the VLD of the SCP were significantly higher in perifovea than those in parafovea (*P* = 0.003). However, the VLD of the DCP did not show significant differences between perifovea and parafovea (*P* = 0.420) (Fig. 1).

The peripapillary VD and parafoveal VD of theSCP showed significant differences among the four quadrants (*P* < 0.001 and *P* < 0.001, respectively). The peripapillary VD had nasal and temporal values significantly lower than the superior and inferior quadrants. The parafoveal VD of the SCP was lowest in the nasal quadrant (Supplementary table S1).

### Correlations between OCTA parameters and systemic factors

The FAZ area of the SCP was positively correlated with duration of DM and diabetic foot (β = 0.178, *P* = 0.047 and β = 0.293, *P* = 0.002, respectively), and negatively correlated with age (β =  − 0.283, *P* = 0.014). The FAZ area of the DCP was positively correlated with gender (male), duration of DM, and diabetic foot (β = 0.197, *P* = 0.033; β = 0.252, *P* = 0.045; and β = 0.304, *P* = 0.002, respectively), and negatively correlated with age and dyslipidemia (β =  − 0.259, *P* = 0.032 and β =  − 0.232, *P* = 0.012, respectively) (Table 3).

**Table 3:** Multiple regression analysis on the predictor of foveal avascular zone area of superficial capillary plexus and deep capillary plexus.

Variables	FAZ area of SCP		FAZ area of DCP	
	β	*P*	β	*P*
Gender (male)	0.140	0.120	**0.197**	**0.033**
Age	− **0.283**	**0.014**	− **0.259**	**0.032**
Smoking (current)	0.020	0.827	− 0.043	0.642
Hypertension	0.062	0.475	0.074	0.402
Dyslipidemia	− 0.161	0.075	− **0.232**	**0.012**
History of CVA	− 0.147	0.099	− 0.082	0.361
History of IHD	− 0.015	0.875	0.001	0.991
DM duration	**0.178**	**0.047**	**0.252**	**0.045**
Insulin use	0.154	0.123	0.135	0.185
Hemodialysis	0.078	0.390	0.100	0.281
Diabetic foot	**0.293**	**0.002**	**0.304**	**0.002**
BMI	− 0.036	0.682	− 0.030	0.739
HbA1c	0.121	0.176	0.070	0.441
Time after PRP	0.185	0.129	0.024	0.843
Anti-VEGF injection	0.077	0.406	0.066	0.484
Lens (pseudophakia)	0.017	0.882	0.050	0.669
Vitrectomy	0.021	0.844	− 0.003	0.979
	R^2^ = 0.352, *P* < 0.001		R^2^ = 0.327, *P* < 0.001	

*FAZ* foveal avascular zone, *SCP* superficial capillary plexus, *DCP* deep capillary plexus, *CVA* cerebrovascular accident, *IHD* ischemic heart disease, *DM* diabetes mellitus, *BMI* body mass index, *PRP* panretinal photocoagulation, *VEGF* vascular endothelial growth factor.

Significant P-values (< 0.05) are in bold.

The parafoveal VD of the SCP was negatively correlated with hypertension, diabetic foot, HbA1c, and time after PRP (β =  − 0.181, *P* = 0.046; β =  − 0.231, *P* = 0.020; β =  − 0.244, *P* = 0.010; and β =  − 0.278, *P* = 0.029, respectively). The CFT was negatively correlated with insulin use, time after PRP, and history of vitrectomy (β =  − 0.273, *P* = 0.005; β =  − 0.292, *P* = 0.014; and β =  − 0.226, *P* = 0.027, respectively) in multiple regression analyses (Table 4). Statistical analysis was also performed for VLD, but the regression models were not appropriate.

**Table 4:** Multiple regression analysis on the predictor of central foveal thickness and parafoveal vessel density of superficial capillary plexus.

Variables	Parafoveal VD of SCP		Central foveal thickness	
	β	*P*	β	*P*
Gender (male)	0.046	0.491	0.018	0.839
Age	0.236	0.056	0.197	0.084
Smoking (current)	− 0.048	0.609	0.024	0.781
Hypertension	− **0.181**	**0.046**	0.052	0.537
Dyslipidemia	0.166	0.076	0.079	0.360
History of CVA	0.131	0.157	0.045	0.596
History of IHD	0.089	0.361	− 0.014	0.881
DM duration	0.123	0.336	− 0.077	0.516
Insulin use	− 0.099	0.342	− **0.273**	**0.005**
Hemodialysis	− 0.006	0.948	− 0.023	0.794
Diabetic foot	− **0.231**	**0.020**	− 0.129	0.156
BMI	− 0.038	0.677	0.035	0.680
HbA1c	− **0.244**	**0.010**	− 0.015	0.864
Time after PRP	− **0.278**	**0.029**	− **0.292**	**0.014**
Anti-VEGF injection	0.112	0.242	0.084	0.343
Lens (pseudophakia)	− 0.123	0.304	− 0.004	0.969
Vitrectomy	0.030	0.781	− **0.226**	**0.027**
	R^2^ = 0.298, *P* < 0.001		R^2^ = 0.395, *P* < 0.001	

*VD* vessel density, *SCP* superficial capillary plexus, *CVA* cerebrovascular accident, *IHD* ischemic heart disease, *DM* diabetes mellitus, *BMI* body mass index, *PRP* panretinal photocoagulation, *VEGF* vascular endothelial growth factor.

Significant P-values (< 0.05) are in bold.

### Correlations between visual acuity and OCTA parameters

Significant correlations were found between BCVA and OCTA parameters. BCVA was negatively correlated with the perifoveal and parafoveal VLD of DCP. However, the CFT, VD, FAZ area, and VLD of the SCP showed no statistically significant correlation (Table 5).

**Table 5:** Correlation of central foveal thickness and OCTA parameters to visual acuity.

	Correlation coefficient	*P* value
Central foveal thickness	− 0.126	0.175
FAZ area of SCP	0.133	0.151
FAZ area of DCP	0.079	0.396
Parafoveal VD of SCP (Average)	− 0.144	0.120
Peripapillary VD (Average)	− 0.123	0.186
Perifoveal VLD of SCP	− 0.110	0.236
Parafoveal VLD of SCP	− 0.114	0.218
Perifoveal VLD of DCP	− 0.348	< 0.001
Parafoveal VLD of DCP	− 0.279	0.002

*FAZ* foveal avascular zone, *SCP* superficial capillary plexus, *DCP* deep capillary plexus, *VD* vessel density, *VLD* vessel length density.

## Discussion

The FAZ area of the DCP was significantly larger than that of the SCP in the patients with quiescent PDR in our study, and the VLD of the DCP were larger than those of the SCP in both perifoveal and parafoveal areas, which are findings similar to those in other DR stages^21,26,27^. Regarding analysis of VLD in the macula, a previous study reported a higher VD of the SCP in parafovea than that in perifovea in both normal patients and patients with NPDR^28^; however, our patients with quiescent PDR showed the opposite result. The VLD was significantly higher at the perifovea than that at the parafovea in the SCP, but there was no topographic difference of VLD in the DCP slab. One possible explanation is that a number of OCTA studies in patients with diabetes have revealed decreased parafoveal VD and enlargement of the FAZ, especially in DCP as DR progressed. Thus, patients with PDR have shown a larger FAZ and a decreased foveal capillary perfusion compared to patients with NPDR and normal patients^15,17,21,26–28^. We assumed that the enlarged FAZ and decreased VD observed in the progression of DR might start in the DCP, followed by the SCP. Therefore, an enlarged FAZ and decreased VD in the SCP could be more prevalent in the advanced stage of DR than the early stage of DR. The enlargement of the FAZ and decrease of VD in the DCP starts from the early stage of DR in the SCP; however, VD in the DCP would already be decreased in both perifovea and parafoveal areas of patients with PDR, and this might have resulted in the absence of topographic differences in the VLD of the DCP in our study. Parafoveal capillaries in the SCP may be damaged as DR progresses to the late stage or past macular edema. Therefore, the angiographic metric in the DCP might be useful to assess early stages of DR, while the VD of the SCP might be more useful to assess and monitor late stages of DR.

Regarding the topographic distribution of VD in macular and peripapillary areas, our study showed significantly lower VD in the nasal and temporal quadrants than the superior and inferior quadrants in both the parafoveal SCP and peripapillary area. Shin et al.^29^ reported that the peripapillary VD and perfusion density in the SCP were lower in the early stage group of DR than in the normal control group; the biggest decline in VD was in the temporal area, whereas VD in the nasal area was preserved in both the peripapillary area and macular SCP. Our study examined patients with PDR who had completed PRP. Photocoagulations were usually delivered one spot size distant from the optic disc in the nasal area, and the temporal macula was spared to avoid an excessive compromise of peripheral vision. We assumed that this PRP treatment caused a greater nasal-quadrant decline in both the peripapillary area and parafoveal SCP in our study. However, VD in the nasal quadrant might be reduced due to PDR; therefore, further research is needed to confirm this result.

We found that some systemic factors were associated with diabetic macular perfusion in patients with quiescent PDR. The variables reflecting the severity of DM, such as presence of diabetic foot and HbA1c, were negatively correlated with the parafoveal VD of the SCP.

Diabetic foot showed correlations with all OCTA parameters (the FAZ area of the SCP and DCP, and parafoveal VD of the SCP) and, in particular, showed a notable *P* value. Since diabetic foot is already associated with abnormal microcirculation, the strong negative correlation of diabetic foot with OCTA parameters is not surprising^30^. Hwang et al. reported that 90% of patients with a diabetic foot had DR, including 55% with PDR; however, only 4.5% of diabetic patients without diabetic foot had DR^31^. Thus, diabetic foot could be used as a surrogate marker for macular perfusion in patients with PDR.

Interestingly, hypertension had a negative correlation with the parafoveal VD of the SCP. In a previous study, hypertension showed no correlation with retinal capillary density^32^. In another report, the VD of the SCP was negatively correlated with systolic blood pressure, and no correlation was found in the VD of the DCP with all systemic factors^33^. The correlation of retinal perfusion with various systemic factors seems to be controversial, therefore further studies are necessary to confirm this.

The CFT was negatively correlated with insulin use, time after PRP, and history of vitrectomy. Insulin use, time after PRP, and history of vitrectomy indirectly reflect poor blood glucose control and long-standing PDR. Foveal thinning is well known to occur in diabetic patients with macular ischemia^11,34^, but time after PRP was the only variable that had a statistically significant association with both parafoveal VD and central foveal thickness in our study. We assume that, with increasing time after PRP treatment, foveal perfusion would diminish and lead to thinning of foveal thickness. Insulin use and vitrectomy may affect foveal thinning in ways other than reduction of macular perfusion.

OCTA parameters reflecting a reduction in macular perfusion were associated with poor visual prognosis in patients with quiescent PDR. Among various parameters, the perifoveal and parafoveal VLD of the DCP showed the negative correlation coefficients, presumably because diabetic macular ischemia of DCP caused the loss of photoreceptor. It is still controversial, but there was a report that ischemia of DCP may be associated with the loss of photoreceptor^35,36^.

Our study has several limitations due to its retrospective, cross-sectional design. First, the manual measurement of FAZ area can introduce variability and reduce consistency. The most important limitation of this study is that it does not accurately take into consideration previous DM control or the extent to which PDR had progressed before PRP. Also, our study does not accurately reflect the effects of PRP on retinal microcirculation over time. Moreover, since patients with macular edema were excluded at the time of the study, we did not examine its effect on macular perfusion state, which could have caused selection bias. Further, large and long-term prospective studies that include patients with macular edema are required to confirm our results and to better understand the effect of systemic conditions on visual outcomes in patients with DR.

In conclusion, diabetic foot, DM duration, time after PRP, and HbA1c were significantly associated with macular perfusion state in patients with quiescent PDR who had completed PRP. Of note, diabetic foot showed the strongest correlation with macular perfusion among the various systemic factors. A large FAZ area and small VD and VLD, especially in the DCP slab, were associated with poor visual outcomes, although the VD of the SCP might be more useful for monitoring PDR. Thus, the possibility of poor vision in diabetic patients with decreased perfusion parameters in the DCP slab and in PDR patients with diabetic foot must be considered.

## Methods

Our retrospective, cross-sectional study was conducted at Hallym University Sacred Heart Hospital, which is a tertiary center in Korea. This study was approved by the Institutional Review Board of Hallym University Sacred Heart Hospital and was performed in accordance with the Declaration of Helsinki. All patients gave informed consent before inclusion in the study.

### Study subjects

The inclusion criteria were patients (1) who attended the retina clinic between July 2019 and October 2019; (2) who had quiescent PDR, which was defined as previously diagnosed PDR that showed regression of neovascularization with no development of neovascularization after PRP for at least 1 year; and (3) who had completed PRP.

The exclusion criteria were patients (1) who were younger than 19 years of age; (2) who had eyes with the presence of macular edema; (3) who had eyes with a history of focal or grid laser treatment; (4) who had eyes with a history of intravitreal corticosteroid injection; (5) who had eyes with cataract or media opacity that would impede image quality; (6) who had eyes with ungradable OCTA images; (7) who had eyes with a history of glaucoma or other retinal disorders (e.g., neovascular glaucoma, retinal vein occlusion, or age-related macular degeneration); and (8) who had eyes with high refractive errors (more than 6 diopters) or astigmatism (more than 3 diopters).

A total of 176 candidates (280 eyes) visited our clinic during the enrollment period; however, 58 patients (113 eyes) were excluded from this study after ophthalmic examination (37 eyes with macular edema, 30 eyes with low image quality, 12 eyes with history of neovascular glaucoma, etc.). Only one eye is randomly selected from the patients. A final total of 118 patients (118 eyes) were eligible for this study.

All participants underwent a thorough ophthalmic examination, including measurements of BCVA, intraocular pressure, manual refraction, slit-lamp biomicroscopy, a dilated fundus exam, ultra-widefield fundus photography by Optos ultra-widefield P200Tx retinal camera (Optos, Marlborough, MA), and OCTA (DRI OCT Triton; Topcon Inc, Tokyo, Japan). The CFT was measured automatically using the built-in software (IMAGEnet6.0, v1.23.15008, Basic License 10).

**Figure 2:**
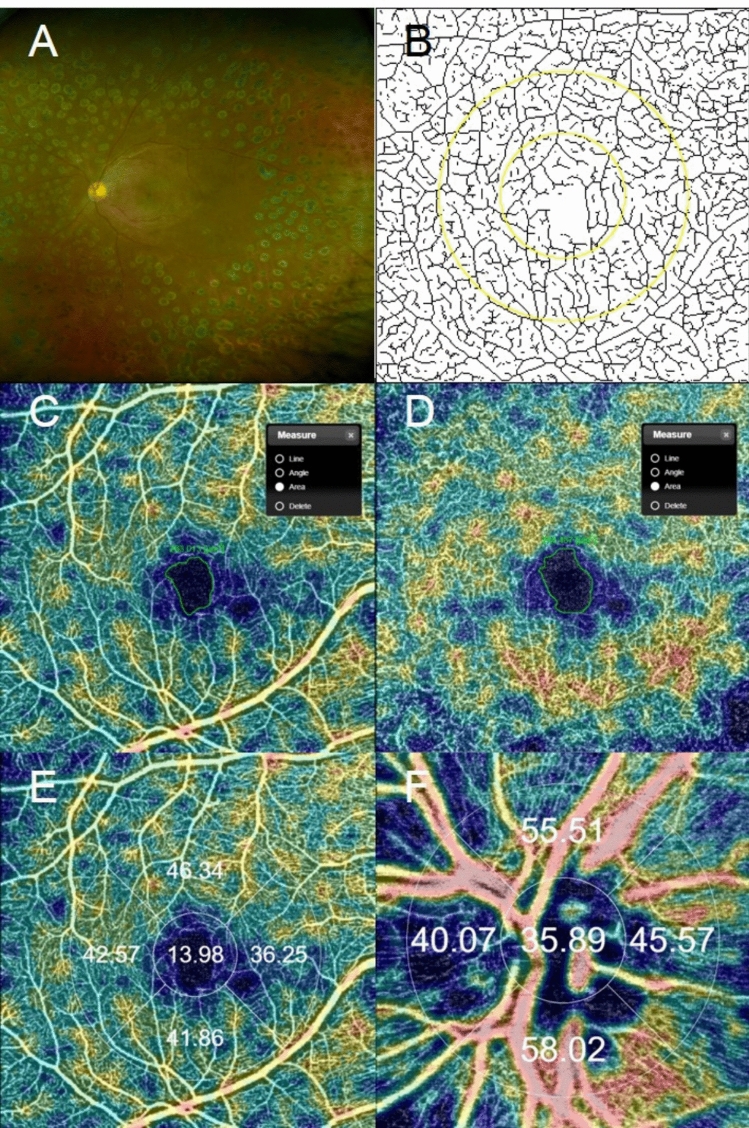
(**A**) Ultra-widefield fundus photography of a patient with quiescent proliferative diabetic retinopathy after panretinal photocoagulation. (**B**) Skeletonized image after binarization. Parafovea was set between a 1.5 mm diameter circle and a 3 mm diameter circle, and perifovea was set between a 3 mm diameter circle and a 4.5 × 4.5 mm square. (**C**) FAZ area of superficial capillary plexus. (**D**) FAZ area of deep capillary plexus. (**E**) Parafoveal vessel density of superficial capillary plexus at macula. (The outer circle has a diameter of 3.0 mm and the inner circle, 1.0 mm, centered at the fovea). (**F**) Peripapillary vessel density (The outer circle has a diameter of 2.5 mm and the inner circle 1.0 mm, centered at the optic disc).

PRP was performed in four sessions in each eye in the order of inferior, superior, nasal, and temporal at an interval of 1 week between sessions. Green or yellow argon laser was delivered through a SuperQuad 160 lens (Volk Optical Inc, Mentor, Ohio, USA) using a slit-lamp adapted photocoagulator (Novus Varia, Lumenis, Salt Lake City, Utah, USA). The individual spots were delivered with a spot size of 200 μm, power of 200 to 400 mW (adjusted to produce grayish white burn), duration of 0.2 s, one spot-size apart of spacing, and 1200 to 1600 of total burns. Treatment area was one disc diameter from the optic disc and the major vessel arcades, and as far as possible in the periphery (Fig. 2A).

### Assessment and definitions of risk factors

All participants underwent a standardized interview. Smoking status (current, ex-, or nonsmoker) and self-reported history of systemic diseases were elicited from the interview. Height, weight, history of cerebrovascular accident and ischemic heart disease, history of DM (including duration, medications, and recent documentation of HbA1c), and history of hemodialysis were self-reported. Diagnoses of hypertension and dyslipidemia were ascertained from documentation of medication use. The presence of diabetic foot was ascertained from reported use of relevant medications or history of relevant surgery and considered as stage 1 to 5 by Wagner’s classification^37^. Body mass index was measured in kg/m^2^.

### OCTA imaging and processing

All participants underwent OCTA using a swept-source OCTA device (DRI OCT Triton; Topcon Inc, Tokyo, Japan) with a wavelength of 1050 nm, an acquisition speed of 100,000 A-scans per second, and axial and transverse resolutions of 7 and 20 μm in tissue, respectively. We used a 4.5 × 4.5 mm scan pattern centered on the fovea and a 3 × 3 mm scan pattern centered on the optic disc. Slabs of SCP and DCP were segmented by the built-in software (IMAGEnet6, v1.23.15008, Basic License 10). SCP was delineated from 2.6 μm below the internal limiting membrane to 15.6 μm below the junction between the inner plexiform and the inner nuclear layers. DCP was delineated from 15.6 to 70.2 μm below the boundary of the inner plexiform and the inner nuclear layers. Only images of sufficient quality, as determined by a signal-strength intensity above 50, were used. Images with a motion artifact or segmentation error were excluded. The OCTA parameters, including the FAZ area, VD, and VLD were evaluated. The FAZ area was manually measured twice with the built-in software (IMAGEnet6) in a masked fashion by one investigator (JK). Results were obtained by analyzing the mean values of two measurements (Fig. 2C,D).

The DRI OCT Triton provided a topographic distribution of the VD in the parafoveal and peripapillary area by automatically calculating the proportion of the measured area with active microvascular supply using the OCTA Ratio Analysis algorithm^38^. The parafoveal VD of the SCP was ascertained at each of the adjacent four sites with the built-in software that employed a modified version of the ETDRS grid with two circles; the outer circle had a diameter of 3.0 mm, and the inner circle had a diameter of 1.0 mm that was centered at the fovea. For the peripapillary VD, circles of 2.5 mm and 1.0 mm diameter centered at the optic disc were used. We analyzed the parameter values in the region between the inner and outer circles; we divided the region into superior, inferior, nasal, and temporal quadrants (Fig. 2E,F). The VD value was then used for the analysis of the topographic difference between the parafoveal and peripapillary SCP. The VD of the DCP could not be analyzed because an automatically calculated VD value was provided only for the SCP in the current generation swept-source OCTA at the time of this study.

For the VLD, the obtained en face images (4.5 × 4.5 mm and 3 × 3 mm) were then exported and analyzed using ImageJ V.1.50 software (National Institutes of Health, Bethesda, Maryland; available at https://rsb.info.nih.gov/ij/index.html) based on the method described by Wen et al.^39,40^ The blood vessels were defined as pixels that had decorrelation values above the threshold level. The second binary form was obtained by skeletonizing the acquired scan into 1-pixel wide vessels, which allowed for the measurement of VLD. The VLD was calculated by measuring the total vessel length in the obtained scan. In order to reduce the effect of the FAZ, we excluded a 1.5 mm diameter circle located at the center of the fovea. Parafovea was set between circles of 1.5 mm and 3 mm diameter, and perifovea was set between the 3 mm diameter circle and a 4.5 × 4.5 mm square. (Fig. 2B).

### Statistical analysis

Multiple regression analysis was performed to evaluate the associations between systemic risk factors and OCTA parameters, as was appropriate for the cross-sectional design of this study. Categorical variables were converted into dummy variables for the analysis. The goodness-of-fit of the multiple regression model was defined as statistically significant at a *P* value < 0.001. R^2^ values are presented to show how well the regression model describes the data. We used standardized beta coefficients to compare the strength of the effect of each variable. An independent *t* test was used to compare the FAZ, and VLD. For the comparison of VD by location, we used one-way analysis of variance (ANOVA) and the Scheffe test for post-hoc comparison. We examined the correlations of visual acuity with central foveal thickness and OCTA parameters using Pearson’s correlation coefficients. All analyses were conducted using SPSS version 24 (IBM Corporation, New York, USA). A *P* value < 0.05 was considered statistically significant.

## Supplementary information

Supplementary Information

## Data Availability

Data supporting the findings of the current study are available from the corresponding author on reasonable request.
