# Supplementary material for: Factors predicting final visual outcome in quiescent proliferative diabetic retinopathy
Source: Sci Rep. 2020 Oct 14;10:17233. doi: 10.1038/s41598-020-74184-9 (PMC7566633; doi:10.1038/s41598-020-74184-9)
Supplement: Supplementary file 1 — Supplementary Information [file 41598_2020_74184_MOESM1_ESM.docx]

**Factors Predicting Final Visual Outcome in Quiescent Proliferative Diabetic Retinopathy**

Jinsoo Kim, In Won Park, Soonil Kwon

Department of Ophthalmology, Hallym University Sacred Heart Hospital, Hallym University College of Medicine, Anyang, Korea

**Financial Disclosures:** The authors have no proprietary or commercial interest in any material discussed in this article.

**Corresponding Author : Soonil Kwon**

**Address :22, Gwanpyeong-ro 170beon-gil, Dongan-gu**

**Hallym University Sacred Heart Hospital, Anyang, Republic of Korea**

**Zip code : 14068**

**Phone number : 82-10- 9075- 5854**

**Fax : 82-31-380-3833**

**Email address :**[**magicham@hallym.or.kr**](mailto:magicham@hallym.or.kr)

**Supplementary table S1.** Vessel density of optic disc and macula in patients.

|  | | Sup | | Inf | | Nasal | Temp | | ANOVA  (P value) | | |
| --- | --- | --- | --- | --- | --- | --- | --- | --- | --- | --- | --- |
|  |  |  |  |  |  |  |  |  |  |  |  |
| Peripapillay VD  (%, mean ± SD) | | 55.4 ± 5.9 | | 56.2 ± 7.4 | | 45.2 ± 7.2 | 46.1 ± 6.4 | | < 0.001 | | |
| Parafoveal VD of SCP  (%, mean ± SD) | | 43.3 ± 6.0 | | 42.3 ± 6.2 | | 39.4 ± 5.0 | 42.0 ± 4.2 | | < 0.001 | | |
| Pairwise comparison | | | | | | | | | | | |
| Sup vs Inf | Sup vs Nasal | | Sup vs Temp | | Inf vs Nasal | | | Inf vs Temp | | Nasal vs Temp |  |
| 0.855 | < 0.001 | | < 0.001 | | < 0.001 | | | < 0.001 | | 0.793 |  |
| 0.586 | < 0.001 | | 0.323 | | 0.001 | | | 0.973 | | 0.005 |  |

Sup = superior, Inf = inferior, Temp = temporal, VD = vessel density, SD = standard deviation, SCP = superficial capillary plexus,

One-way analysis of variance (ANOVA) with post-hoc comparisons using the Scheffe test
